# MIM1 – a selective Mcl-1 protein inhibitor augments the proapoptotic activity of moxifloxacin toward MDA-MB-231 triple-negative breast cancer cells – a preliminary in vitro study

**DOI:** 10.1007/s43440-025-00780-z

**Published:** 2025-09-02

**Authors:** Artur Beberok, Zuzanna Rzepka, Jakub Rok, Marta Karkoszka-Stanowska, Dorota Wrześniok

**Affiliations:** Faculty of Pharmaceutical Sciences in Sosnowiec, Department of Pharmaceutical Chemistry, Medical University of Silesia, Jagiellońska 4, Sosnowiec, 41-200 Poland

**Keywords:** Moxifloxacin, BH3 mimetic – MIM1, Cytotoxicity, Apoptosis, Breast cancer cells

## Abstract

**Background:**

Triple-negative breast cancer (TNBC) is characterized by high invasiveness, high metastatic potential, and poor prognosis. TNBC is not sensitive to endocrine therapy or HER2-targeted treatment, highlighting the need for the development of standardized TNBC treatment regimens. Thus, the development of new TNBC treatment strategies has become an urgent need. MIM1 – BH3 mimetic, which inhibits Mcl-1 antiapoptotic protein, may be an efficacious molecule able to induce apoptosis. Previously, we found that moxifloxacin (MOXI) can modulate the Mcl-1 protein expression. Therefore, in the current study, we assessed the impact of MIM1 and MOXI/MIM1 mixtures on the viability and apoptosis in MDA-MB-231 breast cancer cells.

**Methods:**

The viability of cells was assessed by the WST-1 assay. The proapoptotic activity of the tested compounds was determined by cytometric technique.

**Results:**

The results showed that MIM1 exerted high cytotoxic and proapoptotic potential. In the case of two-component models, we have demonstrated that the use of MOXI and MIM1 mixtures resulted in a significant intensification of both cytotoxic and proapoptotic activity – shown as a modulatory effect on mitochondrial membrane potential, cell cycle distribution, or DNA fragmentation, toward MDA-MB-231 cells when compared with MIM1 or MOXI alone.

**Conclusions:**

We have reported, for the first time, the high proapoptotic activity of MIM1 toward MDA-MB-231 cells pointing to the Mcl-1 protein as an important molecular target. Moreover, the observed potential synergistic mode of action (expressed as a significant enhancement of the induction of apoptosis process) detected for MOXI and MIM1 in the two-component model may provide a new direction for further in vitro and in vivo experiments concerning the role of Mcl-1 protein in the treatment of TNBC.

**Clinical trial number:**

Not applicable.

## Introduction

Breast cancer constitutes one of the most frequently diagnosed cancers among women worldwide. Over the past few years, there has been a steady increase in incidence, particularly evident in highly developed countries. Classification of breast cancers, based on immunohistochemical techniques, involves the expression of the following hormone receptors: estrogen receptor (ER), progesterone receptor (PR), and human epidermal growth factor receptor 2 (HER2) [1]. In recent years, triple-negative breast cancer (TNBC), which responds poorly to drug treatment and accounts for about one-fifth of the overall incidence, has attracted particular attention from the scientific community. TNBC does not overexpress any of the receptors and represents a broad group of diseases with different histological, immunological, and genomic profiles [2, 3]. The mortality rate, high morbidity, strong invasiveness, and resistance to treatment with available pharmacological therapies of triple-negative breast cancer form the basis for the search for targeted therapies that may increase response to treatment.

BCL-2 family proteins, which play a crucial role in the regulation of apoptosis, are suspected to have a major role in the survival of TNBC breast cancer cells. One of the BCL-2 family proteins whose elevated levels are noted in TNBC is the anti-apoptotic protein Mcl-1. Studies reveal that Mcl-1 may be considered an important target for the treatment of TNBC [4]. It was demonstrated that a representative of a compound possessing selective binding and modulatory activity to Mcl-1 protein is BH-3 mimetic - MIM1 (4-((E)-((Z)-2-(cyclohexylimino)-4-methylthiazol-3(2 H)-ylimino)methyl) benzene 1,2,3-triol) [5]. Previously, the presented data showed that MIM1, which possesses the capacity to inhibit Mcl-1 antiapoptotic protein, may be an efficient compound able to induce apoptosis and sensitize melanoma [5, 6] as well as glioblastoma multiforme [7] cells to alkylating chemotherapeutics. It was discovered that the specific Mcl-1 protein inhibitor - MIM1 i/ decreases C32 cells viability, ii/ induces apoptosis (shown as cell-cycle modulatory effect - S-phase arrest, DNA fragmentation as well as redox imbalance – decrease of intracellular GSH level) in amelanotic melanoma cells and iii/ intensifies the proapoptotic properties of dacarbazine, as a result of interactions with Mcl-1 protein [5].

The latest studies concerning fluoroquinolones - a widely used antimicrobial group of drugs- have demonstrated their anticancer activity [8]. Conducting research in this direction, the obtained results of our earlier studies revealed that one of the fluoroquinolones that possesses the proapoptotic effect toward melanoma [9] and breast cancer [10] cells is MOXI. Moreover, based on the in silico and in vitro panel of experiments, we found that this drug exhibits the ability to interact and modulate the Mcl-1 protein expression as a common molecular target with BH3 mimetics. The obtained results from in silico analysis were consistent with the study conducted by Tron et al. [11] where Arg263 of Mcl-1 was shown as an important hot-spot for binding of AZD5991, the Mcl-1-specifc inhibitor. Moreover, we have demonstrated that MOXI can increase the level of Mcl-1 protein in MDA-MB-231 breast cancer cells. Interestingly, the same direction of modulatory effect was confirmed for MIM1, indicating the Mcl-1 protein as a potential molecular target involved in apoptosis induction [5]. The same phenomenon was also demonstrated in the case of ciprofloxacin and C32 amelanotic melanoma cells [12]. This suggests the possibility of fluoroquinolones and Mcl-1 inhibitors synergistic mode of action, thus leading to the induction or strengthening of apoptosis in cancer cells.

The current study is a continuation of our previous study [10] concerning the role of Mcl-1 protein in MOXI cytotoxic and proapoptotic effects toward TNBC cancer cells. Therefore, the purpose of the present study was to determine the effect of MIM1, MOXI alone or in combination, on the viability and apoptosis, assessed by mitochondrial potential breakdown, DNA fragmentation, and cell cycle progression of MDA-MB-231 breast cancer cells.

## Materials and methods

### Cell culture and treatment

Human TNBC breast cancer cells MDA-MB-231, a commonly used TNBC research model, purchased from ATCC (ATCC HTB-26, Manassas, VA, USA), were incubated in a humidified 5% CO_2_ incubator at 37 °C (according to the protocol). The cells were cultured in the recommended medium supplemented with inactivated fetal bovine serum and the following antibiotics: penicillin (100 U/mL), streptomycin (100 U/mL) (cat. 15140122, Thermo Fisher Scientific, Waltham, MA, USA), and amphotericin B (0.25 µg/mL) (cat. 15290018, Thermo Fisher Scientific, Waltham, MA, USA). MDA-MB-231 cells were maintained in DMEM medium (cat. P04-05550, PAN-Biotech, Aidenbach, Germany). Treatment with MOXI (Avelox solution for infusion − 1 bottle of 250 ml containing 400 mg MOXI as hydrochloride, Bayer Healthcare Pharmaceuticals Inc., Germany), MIM1 (cat. 444130, Merck Millipore, Germany) alone or in combination was started 24 h after seeding for breast cancer cells. The analyzed compound solutions were obtained by diluting the stock solutions in a culture medium. The analysis using the WST-1 assay was made for the compound concentrations ranging from 100 µM to 500 µM for MOXI [10] and from 1 µM to 100 µM for MIM1. Other studies, concerning the assessment of apoptosis, were performed for MOXI and MIM1 at concentrations of 100 µM, 500 µM, and 50 µM, 100 µM in one- or two-component model models. Before the experiments, in the preliminary analysis using the spectrophotometric method, we conducted stability tests for the applied compounds. The obtained results showed no changes in the absorption spectra of the tested compounds in the analysed concentration range for individual time points (24 h, 48 h, 72 h).

### Cell viability assessment – WST-1 assay

The viability of MDA-MB-231 cells was detected spectrophotometrically with the use of the Cell Proliferation Reagent WST-1 -1 (cat. 5015944001, Roche GmbH, Mannheim, Germany) that can be reduced in viable cells to formazan dye (dark red) by mitochondrial dehydrogenases. In the conducted analysis, 3 × 10^3^ cells per well were placed in a 96-well microplate in a supplemented growth medium and incubated for 24 h. Then the medium was removed and cells were exposed to the studied compounds in a one- or two-component model for 24 h, 48 h, and 72 h. WST-1 was added to MDA-MB-231 cells cultured in 96-well microplates (10 µL/well) 3 h before the measurement. A microplate reader, Infinite 200 PRO (TECAN, Männedorf, Switzerland), was applied to detect the absorbance at 440 nm and 650 nm as a reference wavelength. In the analysis, control samples were normalized to 100%. All the tested samples were calculated as a percentage of the control.

### Analysis of apoptosis

All the experiments were conducted using the fluorescence image cytometer NucleoCounter^®^ NC-3000™ (ChemoMetec, Lillerød, Denmark). In the performed analysis, MDA-MB-231 breast cancer cells were seeded in T-75 flasks at a density of 2 × 10^6^ cells per flask. Treatment of MDA-MB-231 cells with MOXI and MIM1 alone or in combination began 24 h after seeding. After 24, 48, or 72 h of incubation, the cells were harvested by trypsinization.

### Mitochondrial potential assay

The assessment of mitochondrial membrane potential is connected with the accumulation of a fluorescent cationic dye JC-1 in the mitochondria in a potential-dependent manner. In brief, after the cells’ exposure, the analyzed samples were counted (cytometric analysis with use of Cell Count Protocol) and suspended (in the amount of 1.0 × 10^6^ cells) in 12.5 µL of Solution 7 (JC-1) (cat. 910–3007, ChemoMetec, Lillerød, Denmark) and incubated at 37^°^C for 10 min. In the next step, the MDA-MB-231 cells were centrifuged for 5 min at 400 x g, washed twice with PBS, and resuspended in 0.25 mL of Solution 8 (DAPI) (cat. 910–3008, ChemoMetec, Lillerød, Denmark). In the last step of sample preparation, cells were loaded into 8-chamber NC-Slides A8™ and analyzed immediately with the use of the Mitochondrial Potential Assay protocol. The obtained results, presented as scatterplots, were used to demarcate the percentage of cells with low mitochondrial potential.

### DNA fragmentation assay

After exposure of the cells to MIM1, MOXI used in one- or two-component model, the tested samples were counted (with the use of image cytometry technique) and fixed with 70% ethanol (cold) (24 h at 0–4 °C). In the next step of analysis, the cell samples were washed (PBS), centrifuged, and stained with Solution 3 (cat. 910–3003, ChemoMetec, Lillerød, Denmark, DAPI and 0.1% Triton X-100 in PBS). The performed analysis was conducted using the DNA Fragmentation Assay protocol.

### Cell cycle analysis

After the treatment with MIM1, MOXI used in one- or two-component model, the analyzed samples of MDA-MB-231 breast cancer cells were counted (cytometric analysis) and fixed using 70% ethanol (cold) for at least 24 h at 0–4 °C. In the next step, the cell pellets were centrifuged, washed with PBS, and resuspended in the staining Solution 3 (cat. 910–3003, ChemoMetec, Lillerød, Denmark). The samples were analyzed according to the Fixed Cell Cycle-DAPI Assay protocol.

### Statistical analysis

In all in vitro experiments, mean values were calculated from at least three separate experiments performed in triplicate (*n* = 9) ± standard error (SD). The results were analyzed using GraphPad Prism 6.01 Software. Two-way analyses of variance (ANOVA) were performed, followed by Tukey’s post hoc test. In all cases, the statistical significance was set at p-value < 0.05.

## Results

### Combined treatment of MDA-MB-231 cells with moxifloxacin and MIM1 intensified the cytotoxic effect

**Fig. 1:**
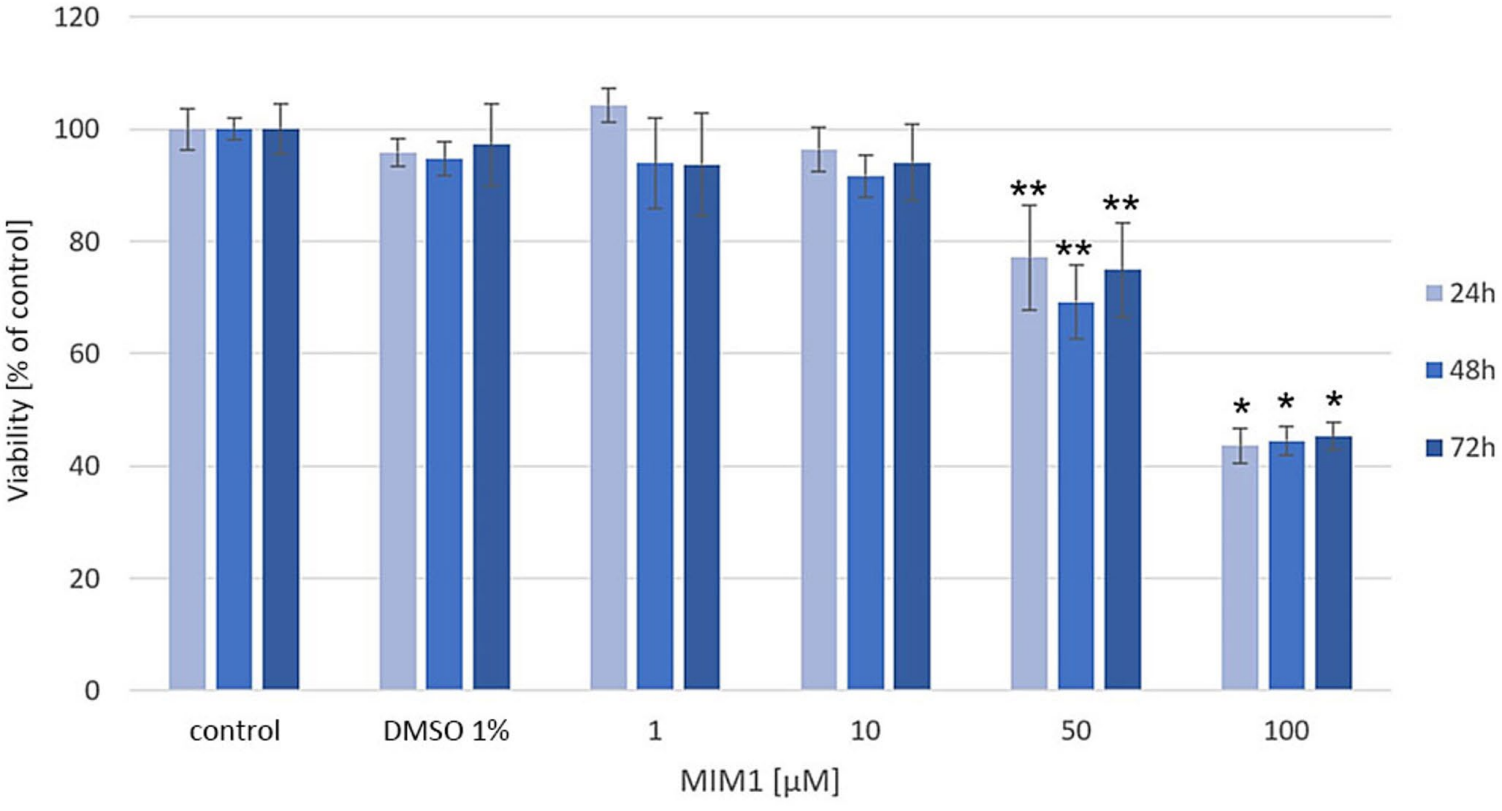
Viability of MDA-MB-231 breast cancer cells cultured in the presence of MIM1 in the range of concentrations from 1–100 µM. The analysis of cell viability was conducted with the use of the WST-1 test. The obtained data are expressed as % of the controls; mean values ± SD (three independent experiments performed in triplicate *n* = 9). Significance was determined using two-way ANOVA followed by Tukey’s post hoc test. * *p* < 0.05 and ** *p* < 0.05 vs. relative control. MIM1 – BH3-mimetic

**Fig. 2:**
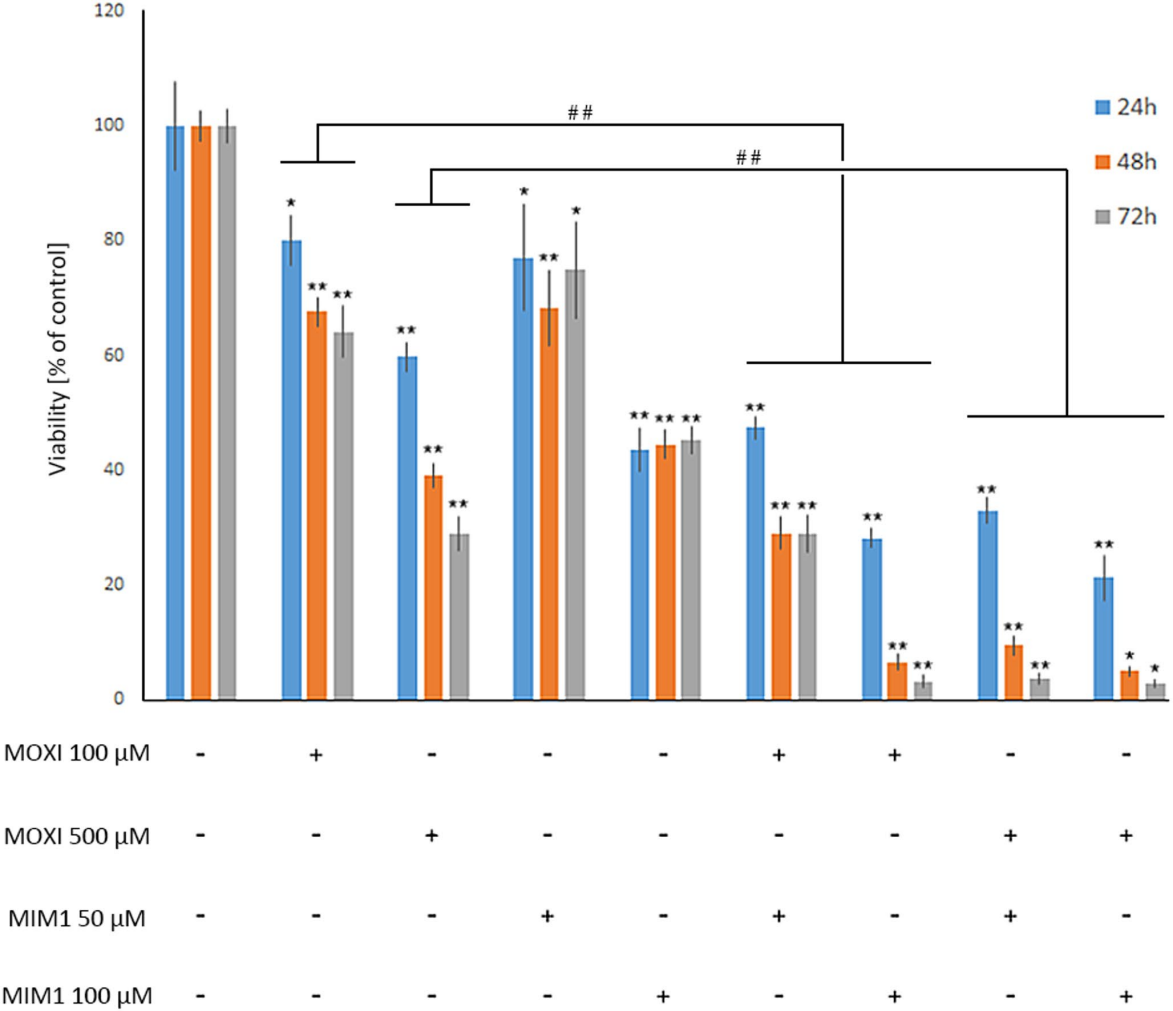
Viability of MDA-MB-231 breast cancer cells cultured in the presence of MOXI, MIM1 in one- and two-compound models. The analysis of cell viability was conducted with the use of the WST-1 assay. The data are expressed as % of the controls; mean values ± SD (three independent experiments performed in triplicate *n* = 9). Significance was determined using two-way ANOVA followed by Tukey’s post hoc test. *p < 0.5 and ** *p* < 0.05 vs. relative control, ## *p* < 0.05 vs. corresponding samples. MOXI – moxifloxacin, MIM1 – BH3-mimetic

Previously, we have demonstrated that MOXI exerts a significant influence on MDA-MB-231 breast cancer cell viability [10]. Based on the obtained data, it was revealed that the studied fluoroquinolone derivatives decreased MDA-MB-231 cells viability by 17–61% for 24 h time of incubation, by 8–89% for 48 h time of incubation, and by 11–98% for 72 h time of incubation in the analyzed range of concentrations from 50 µM to 1000 µM, respectively. In the current study, it was found that the BH3 mimetic – MIM1 also possesses high cytotoxic activity. As presented in Fig. 1, for the analyzed compound concentrations (range from 1 µM to 100 µM) the significant decrease in cells viability was detected in the case of higher MIM1 concentrations – 50 µM (by 23%, 32%, and 25% for 24 h, 48 h and 72 h of incubation, respectively) and 100 µM (by 57%, 56% and 55% for 24 h, 48 h and 72 h of incubation, respectively). A two-way ANOVA revealed a significant effect of concentration (F _4, 120_ = 717, *p* < 0.0001), a significant effect of time (F_2, 120_ = 3.062, *p* < 0.05), and a significant concentration × time interaction (F _8, 120_ = 3.197, *p* < 0.005). Post hoc analysis demonstrated significant differences between the 50 µM and 100 µM MIM1-treated groups compared to groups treated with lower concentrations. Interestingly, the culture of MDA-MB-231 cells with MOXI and MIM1 in a two-component model significantly intensified the cytotoxic effect (Fig. 2). In the studied two-component models – MOXI 100 µM + MIM1 50 µM, MOXI 100 µM + MIM1 100 µM, MOXI 500 µM + MIM1 50 µM and MOXI 500 µM + MIM1 100 µM the analyzed parameter decreased significantly by 53%, 72%, 67% and 79% in the case of 24 h incubation time, by 71%, 93%, 90% and 95% in the case of 48 h incubation time as well as by 71%, 97%, 96% and 97% in the case of 72 h incubation time, respectively. A two-way ANOVA revealed a significant effect of concentration (F _4, 120_ = 4578, *p* < 0.0001), a significant effect of time (F _2, 120_ = 522.1, *p* < 0.0001), and a significant concentration × time interaction (F _8, 120_ = 38.48, *p* < 0.0001). Post hoc analysis demonstrated significant differences between all combined treatment groups compared to controls, with stronger effects observed at higher concentrations and longer incubation times.

### Combined treatment of MDA-MB-231 cells with MOXI and MIM1 augments the proapoptotic effect

**Fig. 3:**
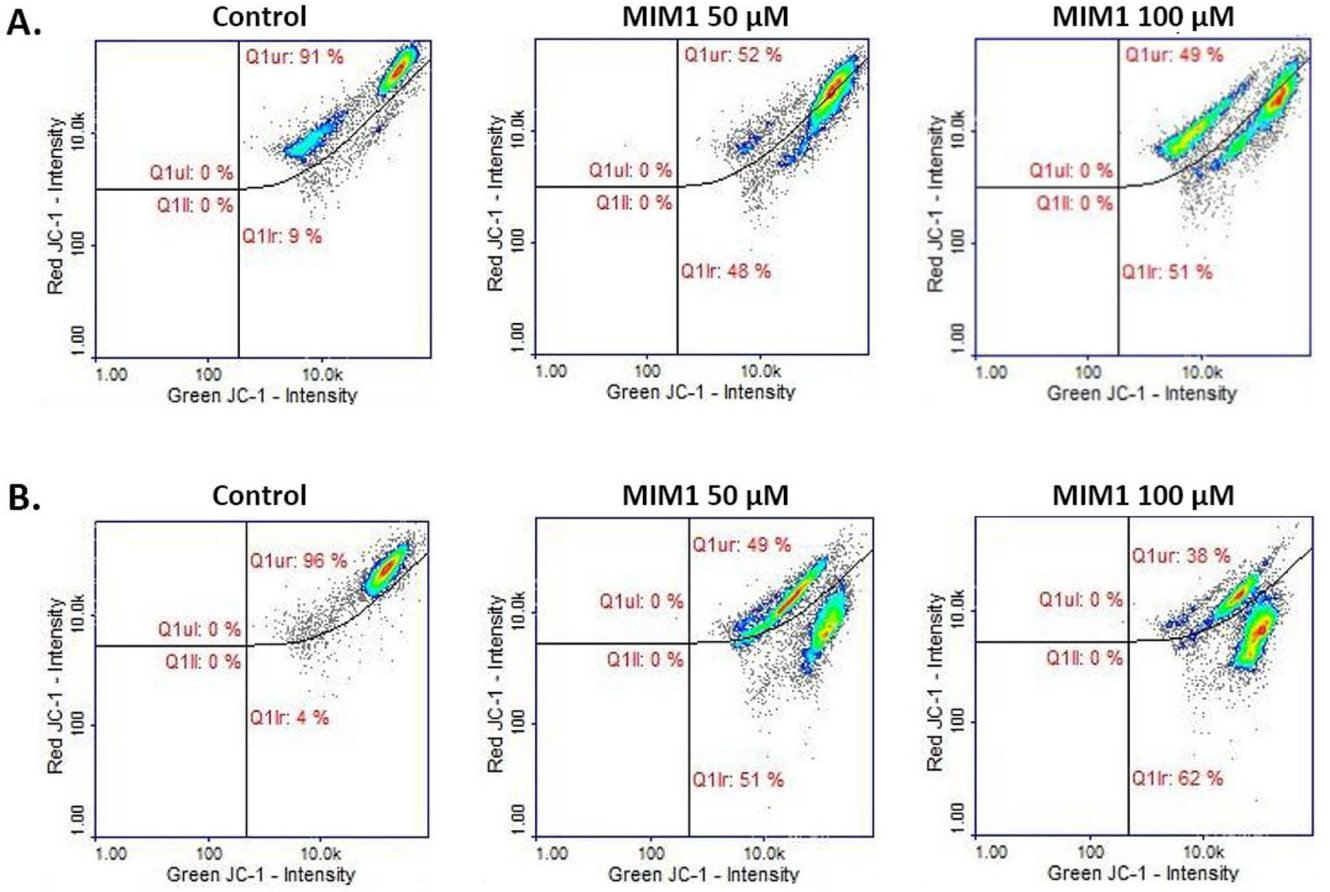
Scatter plots presenting changes in the mitochondrial membrane potential (JC-1 staining) in MDA-MB-231 breast cancer cells treated with MIM1 in concentrations of 50 µM and 100 µM after 24 h (**A**) and 48 h (**B**). The analysis was conducted with the use of image cytometry. The graphs are representative of three independent experiments performed in triplicate (*n* = 9); Q1ur —cells with polarized mitochondria (healthy); Q1lr—cells with depolarized mitochondria (early apoptotic). MIM1 – BH3-mimetic, JC-1 staining - The membrane-permeant JC-1 dye staining for mitochondrial membrane potential changes analysis

In the previously conducted experimental panel experiments, we have pointed out that MOXI induces apoptosis in MDA-MB-231 breast cancer cells via the intrinsic death signalling pathway [10]. When analysing the obtained data for the mitochondrial potential analysis, it was stated that the fluoroquinolone derivative induced significant changes in the mitochondrial membrane potential in the studied cells. This effect was detected especially when MDA-MB-231 breast cancer cells were treated with the drug at the highest analyzed concentration (1000 µM) for 24 h, 48 h, and 72 h, where the percentage of depolarized-early apoptotic cells increased from about 7% (control) to 22%, 74%, and 94%, respectively. Herein, for the first time, MIM1 was found to induce significant alterations in the mitochondrial membrane potential in the analyzed breast cancer cells (Fig. 3; Table 1). For the tested Mcl-1 protein inhibitor concentrations – 50 µM, 100 µM, as well as 24 h and 48 h incubation time, the analyzed parameter significantly increased from about 9% and 4% (controls) to about 48%, 51% and 51%, 62%, respectively. A two-way ANOVA revealed a significant effect of concentration (F _2, 48_ = 40,603, *p* < 0.0001), a significant effect of time (F _1, 48_ = 470.8, *p* < 0.0001), and a significant concentration × time interaction (F _2, 48_ = 1003, *p* < 0.0001). Post hoc analysis demonstrated significant increases in the analyzed parameter for both concentrations compared to controls, with a stronger effect observed after 48 h of incubation. Thus, it may be pointed out that the Mcl-1 antiapoptotic protein could be considered as an important molecular target in the proapoptotic activity of MIM1.

**Fig. 4:**
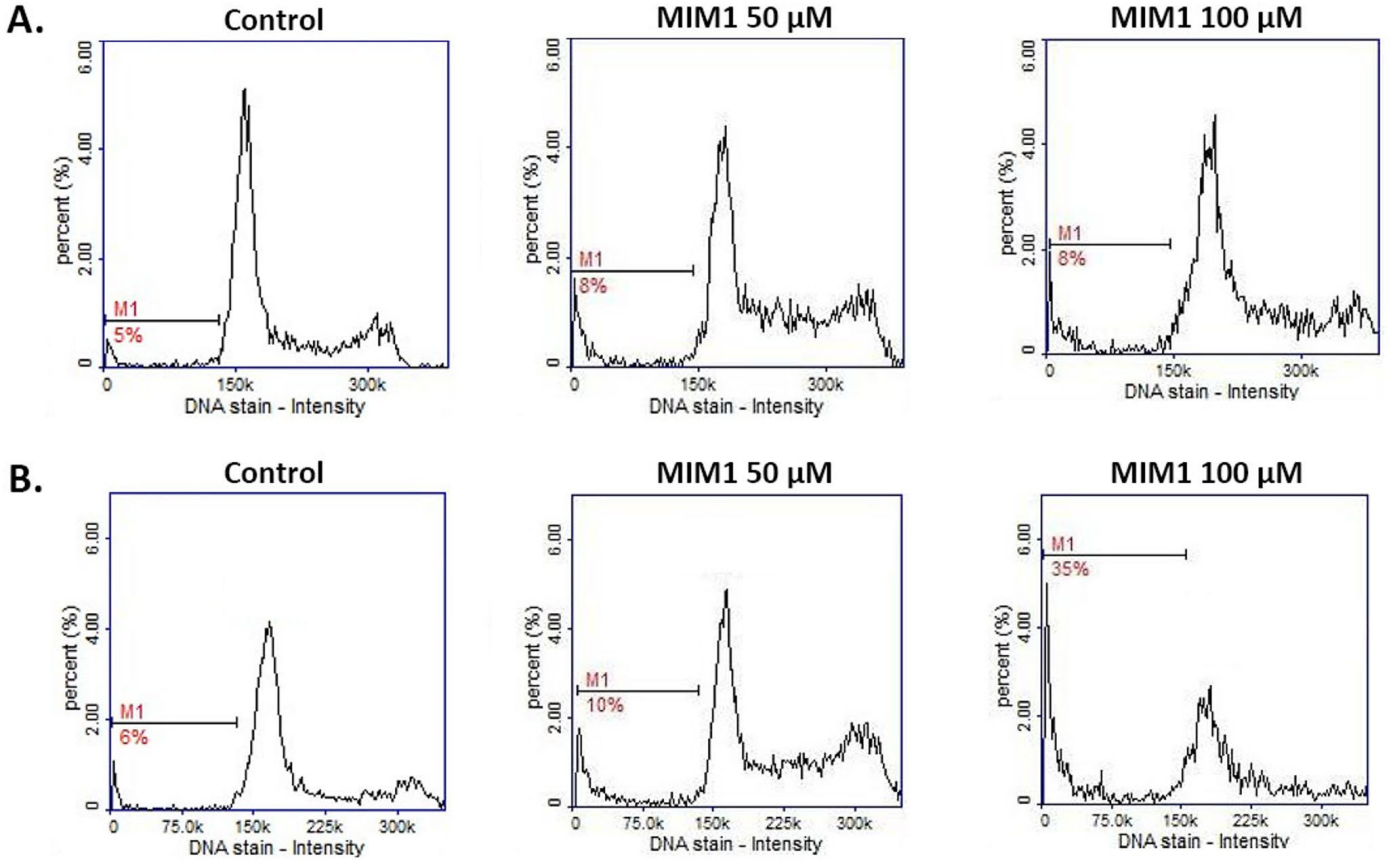
The cytometric analysis of DNA fragmentation (DAPI and 0.1% Triton X-100 in PBS staining) in breast cancer cells treated with MIM1 for 24 h (**A**) or 48 h (**B**). The histograms are representative of three independent experiments performed in triplicate **(***n* = 9**)**; M1—cells with less than 1 DNA equivalent (cells with fragmented DNA). MIM1 – BH3-mimetic

In the group of apoptosis features, DNA fragmentation is considered to be an important apoptotic indicator. DNA fragmentation analysis, as the final step of programmed cell death in MDA-MB-231 cells after MOXI treatment, was the subject of an earlier study [10]. We have revealed that the analyzed drug-induced DNA fragmentation in breast cancer cells, however, this effect was detected only for MOXI concentration 1000 µM for 24 h, 48 h, and 72 h – the percentages of cells having less than one DNA equivalent increased from about 5% (control) to 10%, 46%, and 85%, respectively. The current analysis with the use of MIM1 as an experimental model confirmed the proapoptotic activity of the tested compound (Fig. 4; Table 2). The use of the Mcl-1 inhibitor resulted in DNA fragmentation in MDA-MB-231 cells – the percentages of cells with fragmented DNA were determined at the level of about 5%, 6% (controls), about 8%, 10% (MIM1 50 µM), and about 8%, 35% (MIM1 100 µM) for 24 h, 48 h time of incubation, respectively. A two-way ANOVA revealed a significant effect of concentration (F _2, 48_ = 2363, *p* < 0.0001), a significant effect of time (F _1, 48_ = 2477, *p* < 0.0001), and a significant concentration × time interaction (F _2, 48_ = 1818, *p* < 0.0001). Post hoc analysis demonstrated that the percentage of cells with fragmented DNA was significantly higher in the MIM1 100 µM-treated cells after 48 h of incubation compared to all other groups, including both the control and MIM1 50 µM conditions at corresponding time points. No significant differences were observed between the 24 h and 48 h time points in the control or 50 µM groups. Given the experimental evidence from our study, MIM1 – a selective Mcl-1 protein inhibitor- may be considered a potent apoptosis inducer in triple-negative breast cancer cells.

**Fig. 5:**
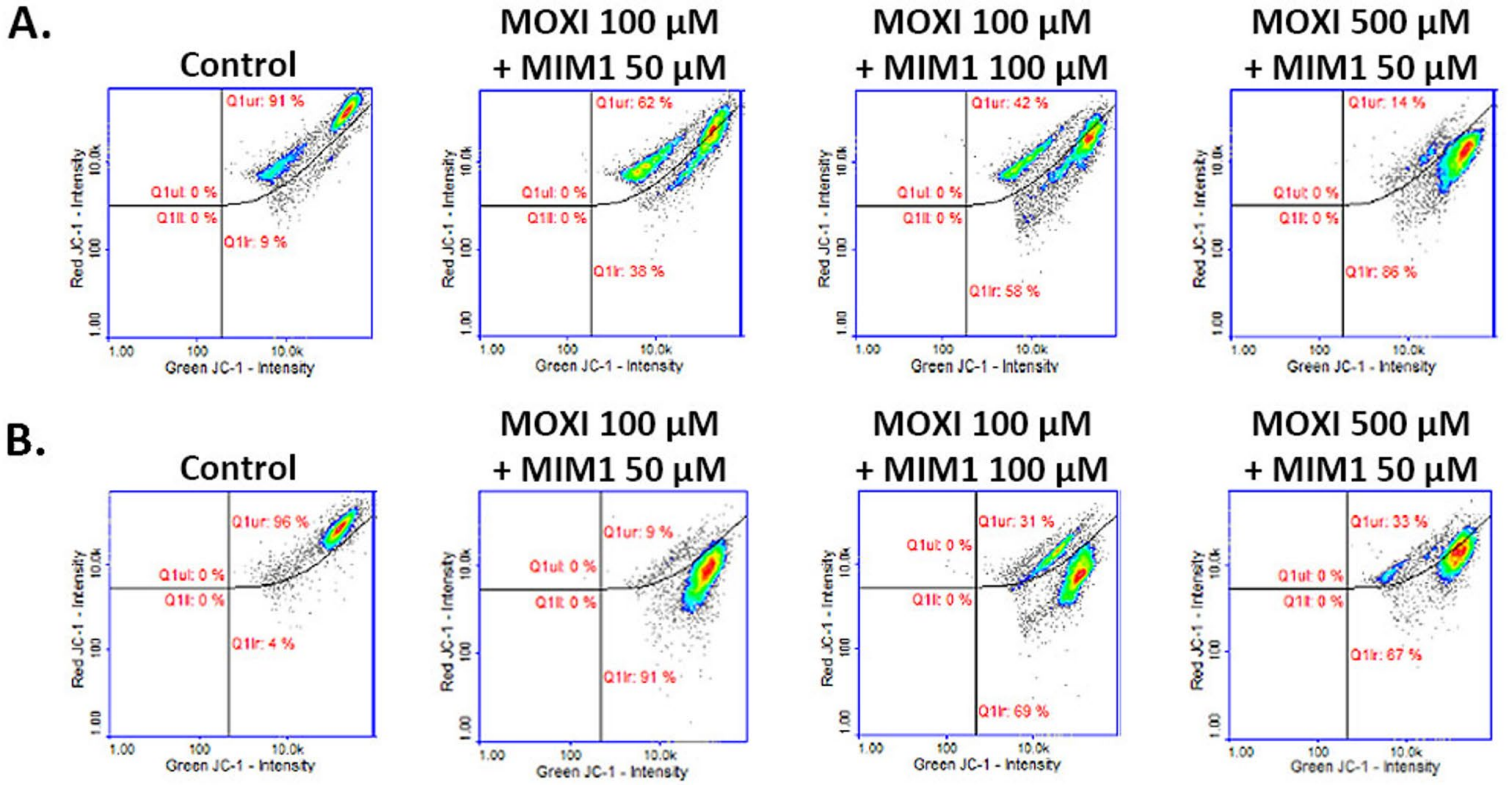
Scatter plots presenting changes in the mitochondrial membrane potential (JC-1 staining) in MDA-MB-231 breast cancer cells treated with MOXI and MIM1 mixture. The analysis was conducted with the use of image cytometry. Cells were exposed to MOXI 100 µM + MIM1 50 µM, MOXI 100 µM + MIM1 100 µM, and MOXI 500 µM + MIM1 50 µM for 24 h (**A**) and 48 h (**B**). The graphs are representative of three independent experiments performed in triplicate (*n* = 9): Q1ur —cells with polarized mitochondria (healthy); and Q1lr—cells with depolarized mitochondria (early apoptotic). MOXI – moxifloxacin, MIM1 – BH3-mimetic

**Fig. 6:**
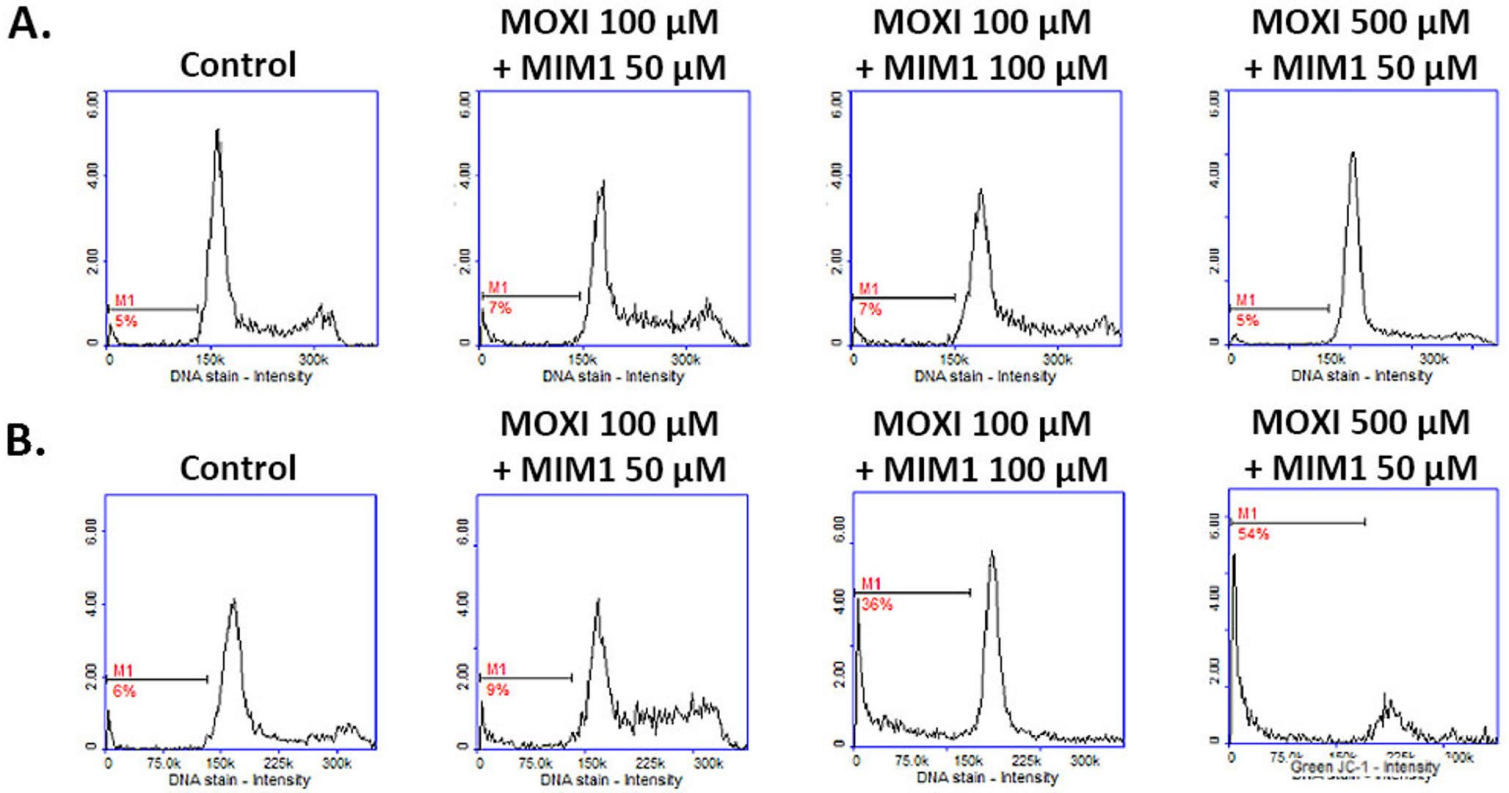
The cytometric analysis of DNA fragmentation (DAPI and 0.1% Triton X-100 in PBS staining) in breast cancer cells treated with MOXI and MIM1 in two-component models for 24 h (**A**) or 48 h (**B**). The graphs are representative of three independent experiments performed in triplicate (*n* = 9); M1—cells with less than 1 DNA equivalent (cells with fragmented DNA). MOXI – moxifloxacin, MIM1 – BH3-mimetic

Interestingly, in the present study, we have found that co-treatment of MDA-MB-231 cells with MOXI and MIM1 intensified both the early and final execution phase of apoptosis (Figs. 5 and 6; Tables 1 and 2). This phenomenon was especially seen after the first 24 h of incubation for MOXI 500 µM + MIM1 50 µM, with an increase of early apoptotic-depolarized cells from about 9–86%. In the case of the exposure of MDA-MB-231 cells to all the studied MOXI/MIM1 models and 48 h incubation time significant increase of depolarized cells was noticed – from about 4–91% for MOXI 100 µM + MIM1 50 µM mixture, from about 4–69% for MOXI 100 µM + MIM1 100 µM mixture, and from about 4–67% for MOXI 500 µM + MIM1 50 µM mixture (Fig. 5; Table 1). A two-way ANOVA revealed a significant effect of concentration (F _3, 64_ = 27,822, *p* < 0.0001), a significant effect of time (F _1, 64_ = 2935, *p* < 0.0001), and a significant concentration × time interaction (F _3, 64_ = 7255, *p* < 0.0001). Post hoc analysis demonstrated that all MOXI/MIM1 combinations caused a significant increase in the proportion of depolarized cells compared to control, with the most prominent effect observed after 48 h of incubation, particularly for the MOXI 100 µM + MIM1 50 µM mixture. For the DNA fragmentation analysis (Fig. 6; Table 2), a significant increase in the analysed parameter was determined for MOXI 100 µM + MIM1 100 µM mixture (from 6 to 36%) and MOXI 500 µM + MIM1 50 µM mixture (from 6 to 54%). A two-way ANOVA revealed a significant effect of concentration (F _3, 64_ = 4650, *p* < 0.0001), a significant effect of time (F _1, 64_ = 14,869, *p* < 0.0001), and a significant concentration × time interaction (F _3, 64_ = 4938, *p* < 0.0001). Post hoc analysis demonstrated that DNA fragmentation was significantly increased after 48 h of incubation in cells treated with MOXI 100 µM + MIM1 100 µM and MOXI 500 µM + MIM1 50 µM compared to control and other treatment groups. This observation indicates that the Mcl-1 protein could be considered a key factor that influences chemosensitivity and programmed cell death.

**Table 1:** The effect of BH3-mimetic MIM1 and MIM1 mixtures with MOXI on mitochondrial membrane potential in MDA-MB-231 breast cancer cells

Analysed sample	Percent of depolarized cells			
	24 h		48 h	
	Mean ± SD	*p*-value (vs. control)	Mean ± SD	*p*-value (vs. control)
Control	9.2 ± 0.4		3.8 ± 0.5	
MIM1 50 µM	47.4 ± 0.8	**< 0.0001**	51.3 ± 0.4	**< 0.0001**
MIM1 100 µM	50.8 ± 0.7	**< 0.0001**	62.4 ± 0.5	**< 0.0001**
MOXI 100 µM + MIM1 50 µM	37.4 ± 0.8	**< 0.0001**	91.6 ± 1.1	**< 0.0001**
MOXI 100 µM + MIM1 100 µM	57.4 ± 0.6	**< 0.0001**	69.6 ± 0.7	**< 0.0001**
MOXI 500 µM + MIM1 50 µM	86.9 ± 1.1	**< 0.0001**	66.8 ± 0.9	**< 0.0001**

Data were analyzed using a two-way ANOVA followed by Tukey’s post hoc test (*n* = 9) MOXI – moxifloxacin, MIM1 – BH3-mimetic

**Table 2:** The effect of BH3-mimetic MIM1 and MIM1 mixtures with MOXI on DNA fragmentation in MDA-MB-231 breast cancer cells

Analysed sample	Percent of cells with fragmented DNA			
	24 h		48 h	
	Mean ± SD	*p*-value (vs. control)	Mean ± SD	*p*-value (vs. control)
Control	5.8 ± 0.4		6.3 ± 03	
MIM1 50 µM	8.4 ± 0.6	**< 0.0001**	10.8 ± 0.9	**< 0.0001**
MIM1 100 µM	8.6 ± 0.8	**< 0.0001**	35.7 ± 1.1	**< 0.0001**
MOXI 100 µM + MIM1 50 µM	7.4 ± 0.5	**0.0015**	9.6 ± 0.8	**< 0.0001**
MOXI 100 µM + MIM1 100 µM	7.3 ± 0.4	**0.0040**	36.7 ± 0.9	**< 0.0001**
MOXI 500 µM + MIM1 50 µM	5.4 ± 0.5	**0.9938**	55.1 ± 1.3	**< 0.0001**

Data were analyzed using a two-way ANOVA followed by Tukey’s post hoc test (*n* = 9) MOXI – moxifloxacin, MIM1 – BH3-mimetic

**Fig. 7:**
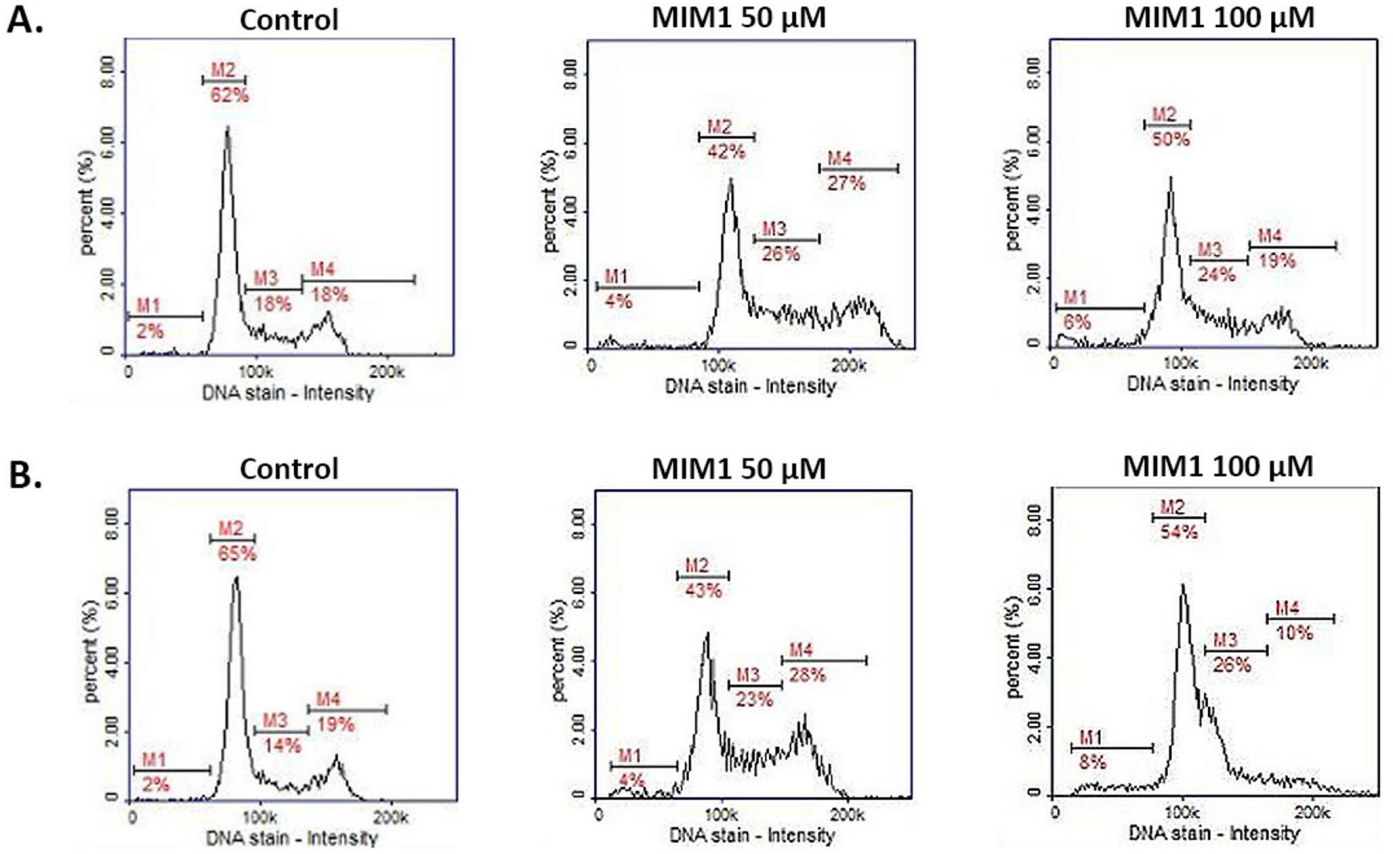
Cytometric analysis of cell cycle distribution (DAPI and 0.1% Triton X-100 in PBS staining) in breast cancer cells treated with MIM1 (50 µM and 100 µM) for 24 h (**A**) or 48 h (**B**). The graphs are representative of three independent experiments performed in triplicate (*n* = 9); M1 – sub-G1 phase, M2 – G0/G1 phase, M3 – S phase, M4 – G2/M phase. MIM1 – BH3-mimetic

**Fig. 8:**
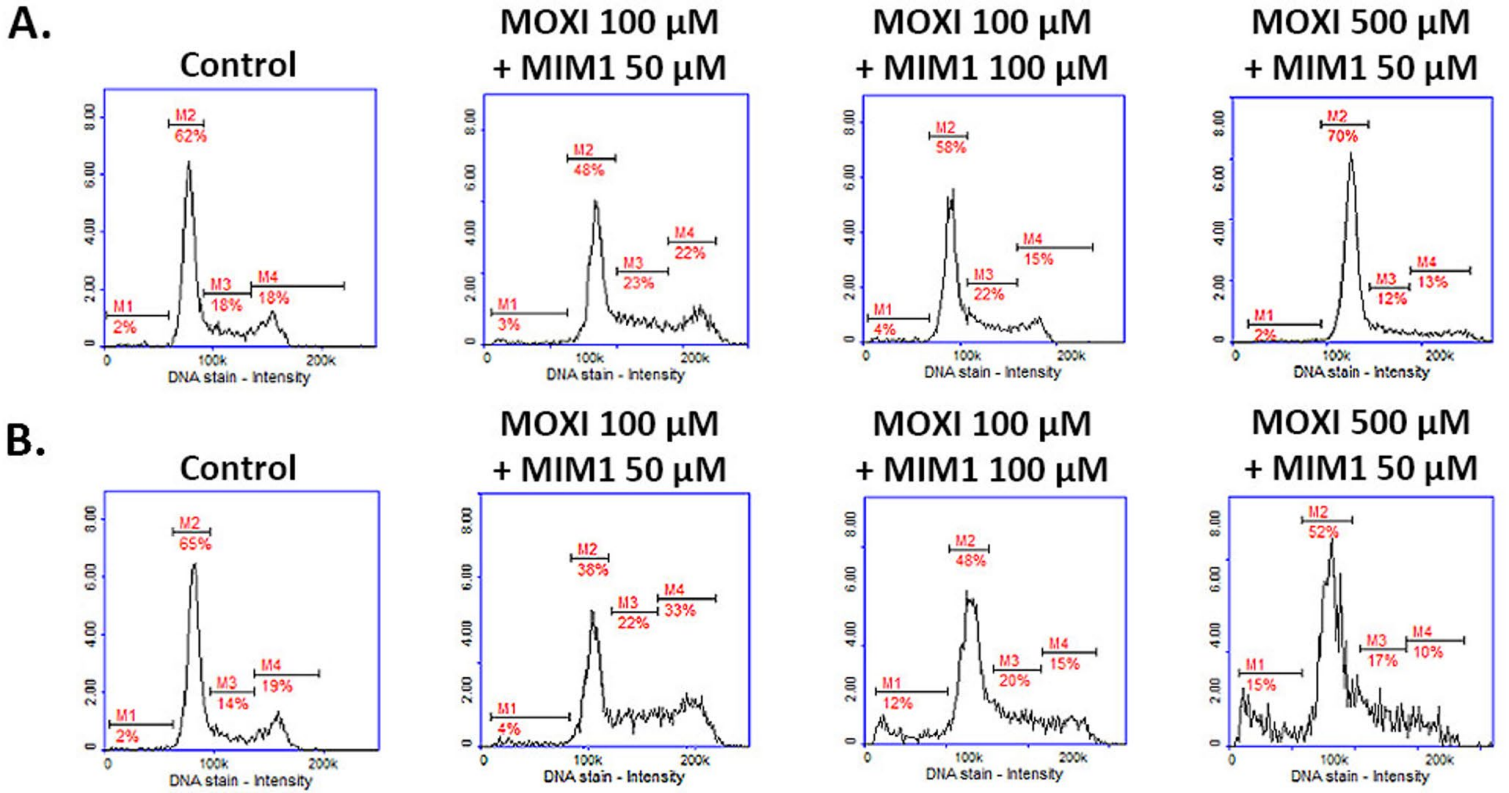
Cytometric analysis of cell cycle distribution in breast cancer cells treated with MOXI and MIM1 mixture. Cells were exposed to MOXI 100 µM + MIM1 50 µM, MOXI 100 µM + MIM1 100 µM, and MOXI 500 µM + MIM1 50 µM for 24 h (**A**) and 48 h (**B**). The graphs are representative of three independent experiments performed in triplicate (*n* = 9); M1 – sub-G1 phase, M2 – G0/G1 phase, M3 – S phase, M4 – G2/M phase. MOXI – moxifloxacin, MIM1 – BH3-mimetic

Apoptosis and cell cycle distribution are closely connected processes that may impact cellular homeostasis. It should be noted that modulation of the cell cycle may both i/prevent or ii/ induce an apoptotic response [13]. Therefore, in the current study, we have assessed the capacity of the tested compounds in one- or two-component models to induce alterations of the cell cycle in MDA-MB-231 breast cancer cells. Previously, it was stated for MOXI that this fluoroquinolone antibiotic, depending on the concentration used, caused both G2/M (500 µM) and S (1000 µM) phase arrest in MDA-MB-231 breast cancer cells. It was stated that after 24 h, the percentages of G2/M and S fractions increased from 16% (control) to 22% and 19% (control) to 31%, respectively. Interestingly, the extension of incubation time up to 48 h significantly increased the population of cells in the sub-G1 phase from 2 to 37% for MOXI in the concentration of 1000 µM [10]. The results of our current study demonstrate for the first time that inhibition of Mcl-1 protein by MIM1 in breast cancer cells induces cell cycle arrest at the G2/M or S phase. Treatment with MIM1 at a concentration of 50 µM for 24 h resulted in an increase in the G2/M population from approximately 18–26% (a two-way ANOVA revealed a significant effect of concentration: F _2, 48_ = 420.7, *p* < 0.0001; a significant effect of time: F _1, 48_ = 38.75, *p* < 0.0001; and a significant concentration × time interaction: F _2, 48_ = 79.09, *p* < 0.0001). Similarly, an increase in the S phase population was observed under the same conditions, from approximately 18–27% (a two-way ANOVA revealed a significant effect of concentration: F _2, 48_ = 420.7, *p* < 0.0001; a significant effect of time: F _1, 48_ = 17.19, *p* = 0.0001; and a significant concentration × time interaction: F _2, 48_ = 21.31, *p* < 0.0001). Post hoc analysis confirmed that these changes were significant compared to controls. Prolongation of incubation time to 48 h resulted in similar alterations in cell cycle distribution (Fig. 7). In the case of combined treatment with MOXI and MIM1 in the two-component model, a significant increase in the percentage of cells arrested in the G2/M phase was observed, rising from approximately 14–22% (a two-way ANOVA revealed a significant concentration × time interaction: F _3, 64_ = 76.29, *p* < 0.0001). Additionally, a notable increase in S phase arrest was detected for the MOXI 100 µM + MIM1 50 µM combination after 48 h of incubation, with the percentage increasing from approximately 19–33% (significant concentration × time interaction: F _3, 64_ = 28.48, *p* < 0.0001). Moreover, at the highest tested concentration (MOXI 500 µM + MIM1 50 µM), a significant accumulation of cells in the sub-G1 phase was observed, with an increase from 2 to 15% (significant concentration × time interaction: F _3, 64_ = 173.2, *p* < 0.0001), indicating enhanced apoptotic DNA degradation (Fig. 8). Post hoc analysis confirmed that these increases were statistically significant compared to the control and other treatment groups.

## Discussion

Based on our earlier in silico and in vitro panel of experiments, we found that MOXI exhibits the ability to interact with the Mcl-1 protein as a common molecular target with BH3 mimetics and suggests the possibility of their synergistic mode of action towards cancer cells with elevated levels of Mcl-1 protein [10]. Mcl-1 exerts its function by binding proapoptotic Bcl-2 proteins and thus preventing apoptosis induction. In the case of breast cancer, overexpression of this protein not only correlates with tumorigenesis but also associates significantly with resistance towards targeted therapy and conventional chemotherapy [4, 14]. Therefore, in the present study, we have assessed the effect of MOXI and MIM1, the Mcl-1 protein inhibitor used in one- as well as two-component models, on MDA-MB-231 breast cancer cells. We have revealed that co-treatment of the studied cells with MOXI and MIM1 potentiates both cytotoxic as well as pro-apoptotic activity when compared with MOXI and MIM1 applied in a one-component model. Therefore, it may be stated that the studied compounds may exert a potential synergistic mode of action.

Based on the obtained results, we have found that MIM1 exerted high cytotoxic activity towards the studied breast cancer cell line. Interestingly. This effect was significantly strengthened when MDA-MB-231 cells were exposed to both MIM1 and MOXI. Respondek et al. [7] have shown that temozolomide demonstrates a significantly lower cytotoxic effect, even in the highest drug concentration (100 µM) and 72 h of incubation time on U87MG glioblastoma cells when compared with MIM1 and MIM1/temozolomide mixture. On the other hand, it was observed that the MIM1/temozolomide mixture exhibited a greater cytotoxic effect on U87MG glioblastoma cells than MIM1 alone, providing for the first time convincing evidence that MIM1, which inhibits Mcl-1 antiapoptotic protein, can sensitize glioblastoma cells to alkylating agents. Moreover, in the study conducted by Valiulienne et al. [15], it was found that metformin had an additive effect on myeloid leukemia cells’ viability when combined with Mcl-1 inhibitor S63845. This effect was also confirmed for TNBC cells – S63845 demonstrated synergistic activity with docetaxel, or in HER2–amplified breast cancer, synergistic activity with trastuzumab or lapatinib [16]. In the current study, to the best of our knowledge, for the first time, we have revealed that co-treatment of MDA-MB-231 breast cancer cells with MOXI and MIM1 resulted in a higher cytotoxic response of the analyzed cancer cells, pointing to the potential synergistic mode of action. This effect was noticed for all the tested mixtures (MOXI 100 µM + MIM1 50 µM, MOXI 100 µM + MIM1 100 µM, and MOXI 500 µM + MIM1 50 µM), especially when cells were exposed to MOXI and MIM1 for 48 h and 72 h. It should be noted that the new anticancer therapy is often associated with a toxic response from normal cells. In previously conducted studies, Kowalska et al. [17] revealed that the use of MOXI in the range of concentrations from 1 µM to 500 µM did not affect the viability of normal human melanocytes. In our current study, with the use of the TNBC in vitro model, MOXI in concentrations of 100 µM and 500 µM resulted in a statistically significant decrease in cell viability by about 20% and 40% in the first 24 h. Therefore, it could be stated that moxifloxacin exerts higher cytotoxic activity in the case of TNBC cells. Moreover, Cohen et al. [18] have demonstrated low cytotoxic activity of MIM1 in the case of normal fibroblasts, pointing to this compound as a safe and effective proapoptotic molecule.

BH3 mimetics have been the subject of many studies as well as clinical trials. Except in a few specific disease types, including hematologic malignancies, including chronic lymphocytic leukemia, as well as blastic plasmacytoid dendritic cell neoplasm, BH3 mimetics applied in a one-component model have not produced high response rates [19–21]. It should be noted that the most assessed form of therapy regarding this group of chemical substances is their combination with current applied anticancer therapies. The main objective for this strategy is that tumors rapidly adapt to applied forms of treatment, including both conventional chemotherapy as well as targeted agents, where persevering cancer cells survive, leading to recurrence. The main mechanism underlying the phenomenon by which chemotherapy may synergize with BH3 mimetics is by lowering the apoptotic threshold of cells [22]. Preclinical data have demonstrated potential synergy in the mode of action between cytotoxic agents, including cytarabine and venetoclax, by i/ enhancing BH3 activity and/or ii/ suppressing MCL1 to induce apoptosis [23]. Consistent with these observations, it was demonstrated that application of venetoclax with chemotherapy was associated with deeper remissions in patients with myelogenous leukemia [24]. In the present study, we have revealed that co-treatment of MDA-MB-231 breast cancer cells with MOXI and MIM1 resulted in apoptosis intensification, expressed as mitochondrial membrane potential breakdown and induction of DNA fragmentation. In the studied experimental models, the observed effect was the sum of the activities of the individual components, pointing out that the MOXI and MIM1 mixture is a very effective apoptosis inducer and thus may exert a synergistic mode of action in the case of TNBC breast cancer cells. Interestingly, we have observed a strong correlation between the results obtained from the viability assay, mitochondrial membrane potential, and DNA fragmentation analysis. The cytotoxic response of MDA-MB-231 cells was more marked after prolongation of incubation time. The effect was especially noticeable for MOXI 100 µM + MIM1 100 µM, MOXI 500 µM, and MIM1 50 µM, where the cell viability decreased by about 90%. At the same time, MDA-MB-231 breast cancer cells treated with MOXI and MIM1 in two-component models for 48 h demonstrated a significant increase in the percentage of depolarized cells, as well as fragmented DNA up to 91% and 54%, respectively.

It is known that the Mcl-1 protein has a potential role in the cell cycle distribution as well as inhibiting apoptosis [25–27]. It was shown for human colon cancer that G2/M cell cycle arrest after Mcl-1 knockdown was caused by decreasing cyclin and CDKs (cyclin-dependent kinases) and increasing CDKI (cyclin-dependent kinase inhibitor) expression. Thus, the results presented in this study point to the induction of significant changes in the cell cycle distribution of MDA-MB-231 cells after MOXI and MIM1 treatment constitutes an important additional factor that promotes apoptosis through Mcl-1 protein inhibition.

## Conclusions

Herein, for the first time, we have demonstrated that MIM1 – the Mcl-1 protein inhibitor- exerts high cytotoxic and proapoptotic activity towards MDA-MB-231 breast cancer cells. Moreover, taking into account our previously published data concerning the capacity of MOXI to interact with Mcl-1 protein [10], we have demonstrated that the analyzed fluoroquinolone derivative and MIM1 used in a two-component model exerted a potential synergistic mode of action towards MDA-MB-231 breast cancer cells. This phenomenon was shown in both cytotoxic and apoptosis induction panels of experiments. In the conclusion, we would like to emphasize that the current study is limited to in vitro experiments, and incorporating in vivo testing would be a logical extension, especially given the promising in vitro results. However, the presented work is a part of a complex and multi-directional design study targeted at fluoroquinolone antibiotics repurposing in cancer therapy with emphasis on the new signalling pathways underlying this effect. Encouraged by the findings from our earlier study [10] and presented in the current manuscript, which demonstrate, for the first time, that the combinatorial therapeutic option of fluoroquinolones with BH3 mimetic MIM1 - an apoptosis inducer with the capacity to inhibit Mcl-1 protein may constitute the potential new therapeutic option for the treatment of triple-negative breast cancer. These findings allow us to answer the possible synergistic proapoptotic effect of fluoroquinolones and MIM1. Therefore, the current study could/ consist of the basis for future both in vitro as well as in vivo experiments, ii/ determine the potential new strategy, and in conclusion, iii/give the future directions in the treatment of cancers for which Mcl-1 protein consists of important molecular targets.

## Data Availability

The data that support the findings of this study are available from the corresponding author upon reasonable request.

## Abbreviations

ER: Estrogen receptor
GSH: Glutathione (reduced)
HER2: Human epidermal growth factor receptor 2
JC-1 staining: The membrane-permeant JC-1 dye staining for mitochondrial membrane potential changes analysis
Mcl-1: Myeloid cell leukemia-1 protein
MIM1: BH3-mimetic
MOXI: Moxifloxacin
PR: Progesterone receptor
Q1ur: Cells with polarized mitochondria (healthy)
Q1lr: Cells with depolarized mitochondria (early apoptotic)
TNBC: Triple-negative breast cancer
